# Identification of microRNA-451a as a Novel Circulating Biomarker for Colorectal Cancer Diagnosis

**DOI:** 10.1155/2020/5236236

**Published:** 2020-08-27

**Authors:** Zhen Zhang, Dai Zhang, Yaping Cui, Yongsheng Qiu, Changhong Miao, Xihua Lu

**Affiliations:** ^1^Department of Anesthesiology, Affiliated Cancer Hospital of ZhengZhou University, Henan Cancer Hospital, ZhengZhou, China; ^2^Department of Laboratory Medicine, The First Affiliated Hospital of Henan University of Chinese Medicine, Zhengzhou, China; ^3^Department of Anesthesiology, Children's Hospital Affiliated to Zhengzhou University, Zhengzhou, China; ^4^Department of Anesthesiology, Affiliated Cancer Hospital of Fudan University, Shanghai, China

## Abstract

**Background:**

Colorectal cancer (CRC) is one of the leading causes of cancer death worldwide. Successful treatment of CRC relies on accurate early diagnosis, which is currently a challenge due to its complexity and personalized pathologies. Thus, novel molecular biomarkers are needed for early CRC detection.

**Methods:**

Gene and microRNA microarray profiling of CRC tissues and miRNA-seq data were analyzed. Candidate microRNA biomarkers were predicted using both CRC-specific network and miRNA-BD tool. Validation analyses were carried out to interrogate the identified candidate CRC biomarkers.

**Results:**

We identified miR-451a as a potential early CRC biomarker circulating in patient's serum. The dysregulation of miR-451a was revealed both in primary tumors and in patients' sera. Downstream analysis validated the tumor suppressor role of miR-451a and high sensitivity of miR-451a in CRC patients, further confirming its potential role as CRC circulation biomarker.

**Conclusion:**

The miR-451a is a potential circulating biomarker for early CRC diagnosis.

## 1. Introduction

To date, colorectal cancer (CRC) is the second leading cause of cancer death among men and women in the United States, and it is also becoming one of the most death-causing cancer worldwide [1]. Previous clinical studies showed that almost 90% of CRC patients who are diagnosed at an early stage have an extended survival rate of 5 to 10 years, whereas only those 12% of the patients can survive when diagnosed at the late stage [2, 3]. This highlights the importance of early diagnosis of CRC. Nowadays, several clinical approaches have been developed for CRC early detection, including fecal occult-blood testing (FOBT), computed tomography (CT or CAT) scan, colonoscopy, and molecular tumor markers [4]. In particular, numerous clinical markers, which include the carcinoembryonic antigen (CEA), cyclooxygenase-2 (COX-2), and thymidyate synthetase (TS), have been identified in the last several decades [5, 6]. The role of biomarker in CRC diagnosis is becoming more important to improve early diagnosis and better prognosis. However, it is still a challenge to create a more accurate, fast, and specific diagnostic and prognostic biomarkers given that CRC is a complex disease with inherently personalized pathologies. Thus, novel molecular biomarkers for CRC diagnosis and prognosis are still highly in demand.

Noncoding RNAs, which include microRNAs (miRNAs), circular RNAs (circRNAs), and long noncoding RNAs (lncRNAs), are investigated for its potential application in diseases diagnosis. Specifically, miRNAs are the most studied noncoding RNAs, which are a set of small endogenous noncoding RNAs that act as the upstream regulators of many biomolecules and pathways. Because they are easy to obtain from body liquids and they relevantly stable in extreme physiological conditions including extreme changes in pH and temperature [7], they can be excellent candidates as circulating biomarkers for complex diseases [8, 9]. Emerging evidence revealed that dysregulation of circulating miRNAs might correspond to tumor genesis and development [10, 11]. For example, relevant studies found that miR-181a can promote angiogenesis in CRC tissues by regulating SRCIN1 so that SRC/VEGF signaling pathway can be promoted [12]. Jin Y et al. observed that miR-32 plays a key role in cell proliferation and migration, as well as in suppressing apoptosis in colon cancer [13]. These studies further enhance the essential roles of microRNAs in cancer and the necessity of identifying novel CRC biomarkers.

**Figure 1:**
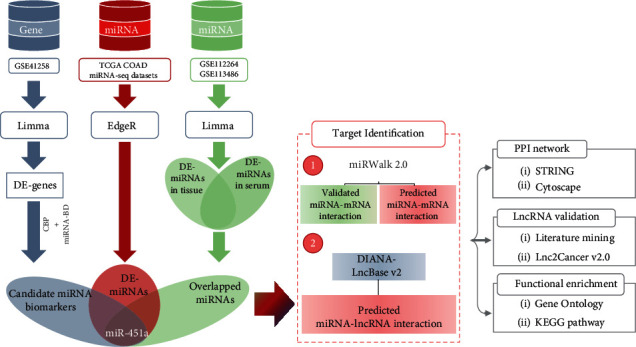
Schematic of the pipeline employed for CRC miRNA biomarker detection.

In this study, we performed an integrated bioinformatics analysis based on multiple microarray and miRNA-seq data to discover novel circulating miRNA biomarkers for CRC diagnosis (Figure 1). Further downstream regulation and function of the candidate biomarkers were also explored. Our research findings could provide new research strategies for CRC biomarker discovery and new insights for CRC diagnosis.

## 2. Materials and Methods

### 2.1. Microarray Data Collection and Processing

CRC relevant microarray data were identified by closely searching the GEO DataSets with the following keywords: “colon∗[Title] AND (cancer [Title] OR carcinoma [Title] OR tumor [Title]) AND “Homo sapiens”[porgn: txid9606].” Furthermore, filters were set to “Expression profiling by array” and “Non-coding RNA profiling by array.” Three publicly available microarray datasets on CRC versus normal colon tissues and serum were downloaded, where GSE41258 is the gene expression array data and GSE112264 and GSE113486 are the microRNA expression array data. All data were downloaded in raw data format. The detailed information on the three datasets is shown in Table 1.

For GSE41258, probe sequences were mapped using the miRBase to obtain the unified name [14]. The expression levels of genes with multiple probe IDs were replaced by their average probe density. Probes with blank gene names and multiple gene names were removed.

### 2.2. Differentially Expressed Genes and microRNA Extraction

In this study, the Limma package was utilized for differentially expressed genes (DE-genes) extraction from GSE41258 [19]. All data were firstly normalized by using “normexp” method, with an offset value of 0. The background-subtracted data were normalized through “quantile” algorithm. All data were then processed by calculating the averages of each miRNA for further statistical analysis. The Student *t*-test was applied to calculate the significant differences (*P* value). Fold changes were calculated according to each gene expression level, comparing between cancer and control group. Final DE-genes were determined using two cutoff criteria: *P* value < 0.05 and ∣ fold change  | >2.

To obtain the differentially expressed microRNAs (DE-miRNAs), two datasets were processed using the Limma package: DE-miRNA screening criteria were the same as what we set in Limma analysis. We then made an overlap between these two DE-miRNA sets. Those differentially expressed miRNAs in both datasets were selected for further validation.

### 2.3. TCGA miRNA-Seq Data Analysis

The microRNA sequencing (miRNA-seq) data were acquired from The Cancer Genome Atlas (TCGA) and filtered using the following strategy: primary site: colon; project: TCGA-COAD; disease type: adenomas and adenocarcinomas; experimental strategy: miRNA-seq; and data type: miRNA expression quantification. A total of 388 microRNA sequencing data with 371 cases were included in our study. One recurrent tumor sample and one metastatic sample were removed. Meanwhile, samples that showed treatment history or did not provide any detailed treatment information were also excluded based on their annotation files. Hence, 329 tumor miRNA-seq datasets and 6 healthy datasets were finally obtained to further investigate DE-miRNAs. The EdgeR was employed for expression analysis [20]. For DE-miRNA cutoff criteria, samples were grouped based on *P* value < 0.05 and ∣ fold change  | >2.

### 2.4. Biomarker Prediction

To identify potential candidate microRNA biomarkers, we used a public microRNA biomarker discovery tool named miRNA-BD, which is based on the DE-genes we acquired from Section 2.2 [21]. A human miRNA-mRNA interaction network was set as default. Two built-in feature parameters, namely the number of single-line regulation (NSR) and transcription factor percentage (TFP), were used here for biomarker identification. The thresholds of NSR and TFP were both set at 2, and the cutoff criteria of *P* value of these two parameters were set at 0.05. In order to improve our ability to discover more convincible CRC biomarkers, we also set a CRC-specific parameter, which is the CRC biomarker percentage (CBP). Relevant studies showed that microRNAs with more target genes are disease-associated or disease biomarkers, and these microRNAs are more likely to serve as disease-specific biomarkers [8]. The formula of the calculation is as follows:

(1) $$\begin{array}{c} \text{CBP}=\frac{\alpha }{\beta }. \end{array}$$

Here, *α* stands for the number of target CRC-associated genes or biomarkers and *β* is the total targets of the microRNAs. As we already acquired DE-miRNAs from miRNA microarray and miRNA-seq analysis, a trioverlap was made to identify more robust miRNA biomarkers.

### 2.5. Identification of Target Genes of miR-451a

The target genes of miR-451a were derived from two online platforms: the miRTarBase and miRWalk 2.0 [22, 23]. First, we searched the hsa-miR-451a in miRTarBase, which provided interactive information about experimentally validated miRNA-mRNA interaction. Further expansion of the target genes was conducted in the miRWalk 2.0, where we both examined experimentally the validated miRNA-mRNA interaction and computational predicted miRNA-mRNA interaction. The 4 algorithms, miRWalk, miRanda [24], RNA22 [25], and Targetscan [26] were used for prediction. Genes that were predicted to interact with miR-451a by a certain algorithm were labeled as 1 out of 4. A threshold of 2/4 was utilized for predicted target gene screening.

### 2.6. Identification of Target Long Noncoding RNAs of miR-451a

DIANA-LncBase v2 was used for miRNA-lncRNA interaction validation and prediction [27]. Experimentally validated lncRNAs, which are regulated by miR-451a, were selected. A threshold of 0.7 was set to screen out lncRNAs, which are predicted to be regulated by miR-451a in colon tissue. To examine CRC-associated lncRNAs, Lnc2Cancer 2.0 database was employed for lncRNA data mining [28] and a final overlap was made between CRC-associated lncRNAs and target lncRNAs of miR-451a.

### 2.7. Functional Enrichment Analysis

To investigate further the role of miRNA-451a, two enrichment analyses, namely gene ontology and pathway analysis, were performed to validate the association between target genes of miR-451a and CRC. The Search Tool for the Retrieval of Interacting Genes (STRING) was used for gene ontology annotation and Kyoto Encyclopedia of Genes and Genomes pathway enrichment [29, 30]. Terms and pathways with *P* value < 0.05 were considered as significantly enriched items. The top 20 most significantly enriched pathways and top 10 most enriched terms were selected, and the association between these items and CRC were further validated through literature mining.

### 2.8. Protein-Protein Interaction Network Analysis

STRING online tool was applied to examine the protein-protein interaction (PPI) patterns of target genes of miR-451a. All target genes, including experimentally validated genes and computational predicted genes, were submitted to STRING for analysis. A combined score of >0.4 was set as the cutoff criterion. The PPI network data generated by STRING was further loaded into Cytoscape for network analysis. CytoHubba, a plug-in tool in Cytoscape, was used for network degree, betweenness, and closeness calculation [31]. The top 10 gene nodes were selected as significant hub genes. Functional modules were predicted using the Molecular Complex Detection (MCODE).

## 3. Results

### 3.1. Identification of Differentially Expressed Genes

**Figure 2:**
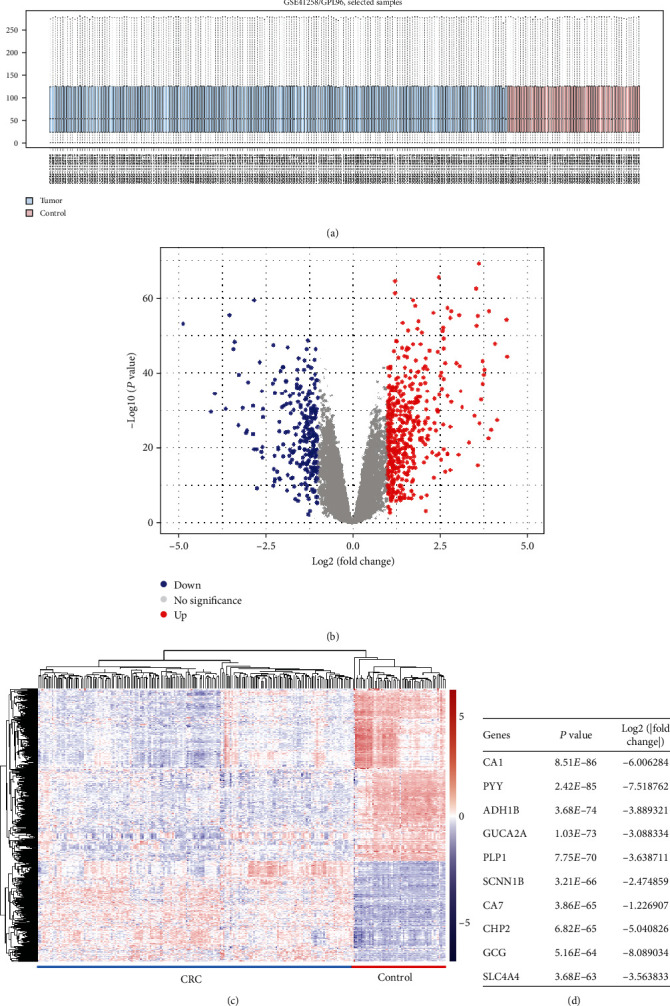
Results of DE-gene analysis. (a) Boxplot of data normalization for GSE41258. Boxes in blue are tumor group and boxes in red are control group. (b) Volcano plot of DE-genes. *X* axis is log2(fold change) and *Y* axis is -log10(*P* value). Significantly downregulated and upregulated genes are painted in blue and red, respectively. (c) Heatmap of DE-genes. (d) Top 10 most significant DE-genes.

To obtain the differentially expressed genes in CRC, we performed a microarray analysis on GSE41258 obtained from GEO DataSets. Data was firstly normalized using the Limma package (Figure 2(a)), and the eBayes algorithm, which was integrated into Limma package, was applied for DE-gene detection. A total of 707 differentially expressed genes were detected through two thresholds' screening: *P* value < 0.05 and log2(∣fold change∣) > 1. Among these genes, 447 were downregulated in primary tissues, while 260 genes were upregulated. The expression pattern of the 707 genes and the top 10 most significant DE-genes are shown in Figures 2(b)–2(d).

Furthermore, literature validation was conducted for the top 10 most significant DE-genes. We noticed that most of the relevant studies revealed potential mechanisms wherein the DE-genes are involved in colon tissue mutation and tumorigenesis. In particular, carbonic anhydrase 1 (CA1) has been implicated as a marker for colon epithelium differentiation [32]. Ghaleb et al. observed that the deletion of Klf4 will lead to a downregulation of CA1, which is highly expressed in colorectal cancer cells [33, 34]. In our analysis, we found a significant upregulation of Klf4 (*P* value = 4.45*E*-42 and log2 (∣fold change∣) = 2.037831). Our data enhanced the relationship between Klf4 and CA1, indicating that the two are most likely coexpressed in colorectal cancer cells. Other genes, such as ADH1B [35], GUCA2A [36], SCNN1B, and CHP2 [37, 38], were also reported to play a role in CRC cell differentiation and tumorigenesis. Taken these results together, our analysis identified DE-genes that are involved in different stages of CRC. These results indicate the demands of cancer cells for quick proliferation, tissue invasion, and metastasis. Hierarchical clustering of DE-genes showed a well-distinguished pattern between primary tumor tissues and healthy colon tissues, suggesting the possibility of selecting features for CRC diagnosis.

### 3.2. Differentially Expressed miRNA Detection and Biomarker Selection

**Figure 3:**
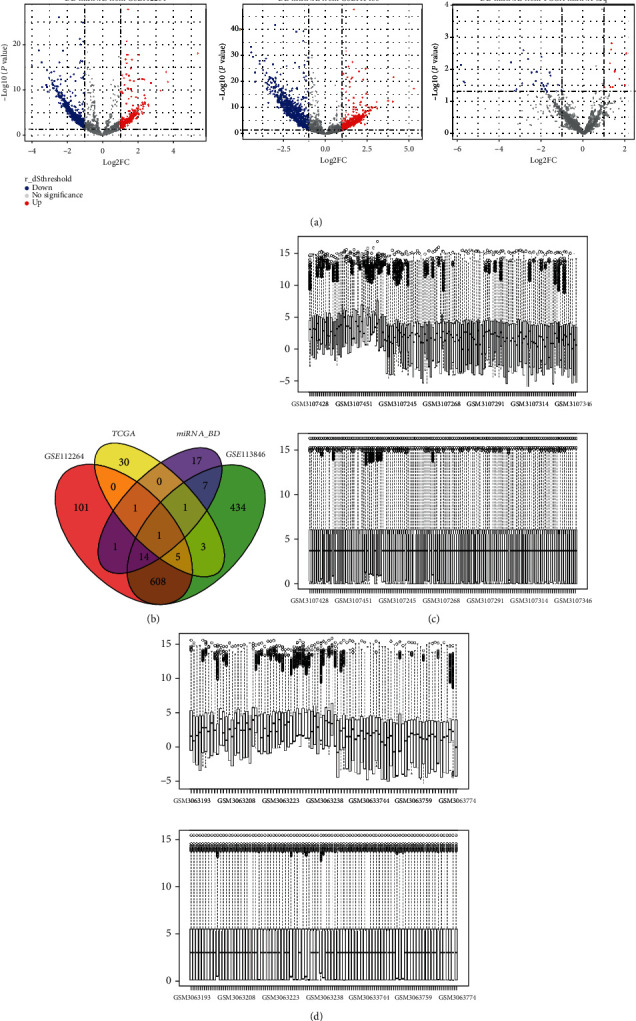
Results of DE-miRNA analysis. (a) Volcano plots for GSE112264, GSE113486, and TCGA miRNA-seq data. (b) Venn plot showed the overlapping results of these DE-miRNAs and candidate biomarkers from these 4 datasets. (c) Boxplot showed the data distribution of GSE113486 before and after normalization and (d) showed that of GSE112264.

To build a CRC specific miRNA-mRNA interaction network, we obtained both miRNA expression microarray data and miRNA-seq data from Geo DataSets and TCGA, respectively. Here, 730 DE-miRNAs with 456 downregulation and 331 upregulation were detected from GSE112264 and 1073 DE-miRNAs with 605 downregulation and 274 upregulation were detected from GSE113486 using the Limma analysis. In miRNA-seq analysis, 40 DE-miRNAs, in which 11 are downregulated and 29 are upregulated, were obtained. The detailed expression information is shown in Figure 3.

Meanwhile, with the DE-genes obtained from Limma DE-gene analysis, a CRC-specific miRNA-mRNA regulatory network was constructed. Then, we performed a miRNA biomarker prediction through miRNA-BD, in which we included the CBP index for more precise prediction. In total, 41 candidate miRNA biomarkers were produced. Among them, 30 miRNAs (73%) were reported to involve in CRC genesis and metastasis. For example, Chai et al. found that miR-223-3p was upregulated in colon cancer [39] and its expression enhancement could cause the suppression of cell apoptosis. Kim et al. found that the expression of miR-590-5p is significantly higher than that in their matched primary CRC [40]. Particularly, by validating the Colorectal Biomarker Database [5], 8 microRNAs (miR-371a-5p, miR-218-5p, miR-21-5p, miR-22-3p, miR-96-5p, miR-150-5p, and miR-200c-3p) have been reported to be useful in CRC diagnosis, treatment, and prognosis biomarkers. These results not only suggest the potential miRNA biomarkers for further investigation but also reveal the accuracy and robustness of miRNA-BD model, which makes our results more convincing.

After overlapping the DE-miRNAs with candidate miRNA biomarkers from miRNA-BD, only one miRNA, which is miR-451a, was identified as the candidate biomarker. Interestingly, miR-451a is downregulated in CRC tissues (with *P* value = 0.0288 and logFC = ‐1.35) but upregulated in human sera (with *P* value = 1.78*E*-10, *P* value = 4.10*E*-13, and logFC = 6.62and logFC = 6.45), indicating changes on gene expression levels regulated by miR-451a between primary tumor tissue and serum. Relevant studies confirmed the expression pattern of miR-451a in primary tumor tissue. Mamoori et al. noticed that the overexpression of miR-451a in colon cancer cells has negative influence on cell proliferation and may increase cell apoptosis [41]. They found that miR-451a results in decreased expression of Oct-4, Snail, and Sox-2 in CRC tissues, among which Oct-4 and Sox-2 are markers of stem cells. Moreover, these two genes are involved in CRC development [42, 43]. Snail is a marker of epithelial-mesenchymal transition (EMT). Meanwhile, Li et al. demonstrated that miR-451a may increase the expression of FoxO3, leading to the downregulation of Ywhaz protein and further inhibition of CRC growth [44]. These findings revealed a tumor suppressor role of miR-451a in CRC primary tumor tissue. In summary, although the regulatory mechanism of miR-451a in human sera is still unclear, miR-451a has a potential role as a circulating biomarker for CRC.

### 3.3. Downstream Target Validation

**Figure 4:**
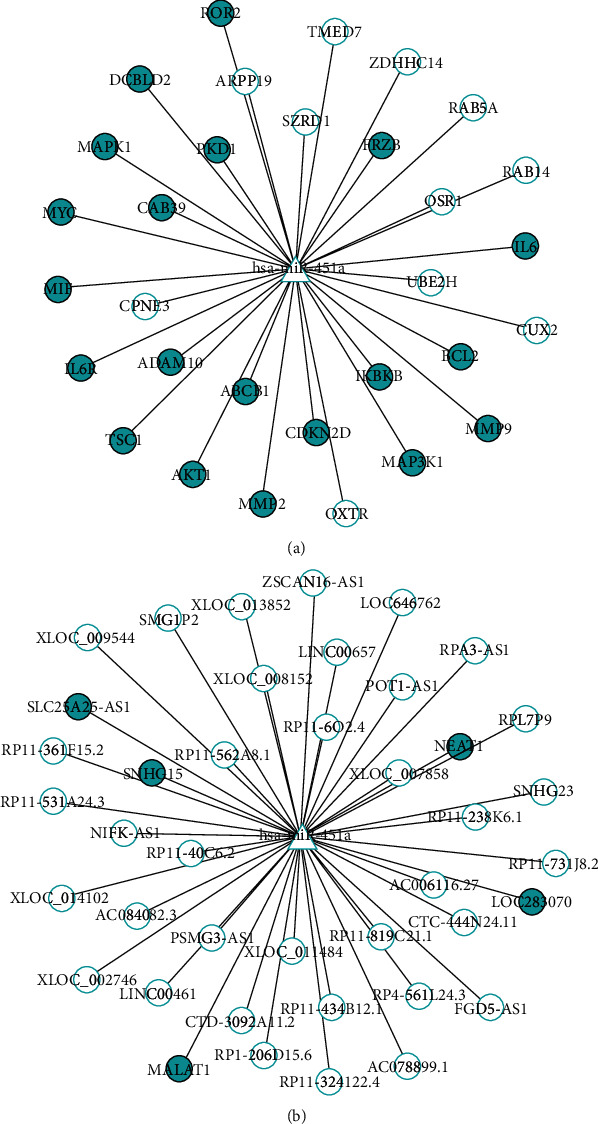
Downstream targets of miR-451a. (a) Experimentally validated mRNA targets of miR-451a. miR-451a was labeled in triangle, and circles here are the mRNAs regulated by miR-451a. Blue solid circles are CRC-associated mRNA (b) LncRNA targets of miR-451a. Blue solid circles are CRC-associated lncRNAs.

To investigate the functional role of miR-451 in CRC, we examined the downstream targets of miR-451a, including mRNAs and lncRNAs. For mRNA target identification, we applied miRWalk 2.0 for both experimental validated targets and computational predicted targets, as well as miRTarBase for experimental validated targets. A total of 24 and 31 validated targets were found from miRWalk and miRTarBase, respectively. In total, 31 experimental validated targets were obtained after a combination of these two results. The regulatory network of miR-451a and these mRNA targets are shown in Figure 4(a). Among these targets, 20 of them (65%) were reported to be involved in CRC tumorigenesis, development, and metastasis. For example, a well-known gene named ROR2 that is involved in both canonical and noncanonical signaling pathways, such as Wnt signaling pathway, was reported to be associated with CRC [45]. The ROR2 protein is a transmembrane receptor for Wnt noncanonical pathway activation. Recent study revealed that the noncanonical Wnt target genes are dependent on ROR2 [46]. Lara et al. also demonstrated that ROR2 is repressed by aberrant promoter of hypermethylation in CRC tissues [47]. Another well-studied gene called MAPK1, which is regulated by miR-451a, was reported recently to be upregulated with the inhibition of miR-145 in CRC tissues [48]. Upregulation of MAPK1 is associated with the promotion of cancer cell proliferation and differentiation [49]. These validated data further strengthen the close relationship between miR-451a and CRC.

MiRWalk was also used to predict potential mRNA targets that are regulated by miR-451a. Under the threshold of 2 as described in methods, a total of 1132 candidate mRNAs were obtained. Most of the predicted mRNAs were reported previously to be involved in CRC, including DISC1, EREG, PPARA, and SYNJ2 [50–53].

The regulatory network of miR-451a and lncRNA targets was built based on the data we acquired from the DIANA-LncBase. We obtained 38 lncRNAs that were validated by immunoprecipitation assay. In addition, 3 lncRNAs were detected through the prediction module of this tool. The miRNA-lncRNA regulatory network of miR-451a is presented in Figure 4(b). We also examined the lncRNAs and demonstrated its association with CRC using the Lnc2Cancer database and 208 lncRNAs selected. By overlapping the CRC-associated lncRNAs and lncRNA targets, 5 of the targets were finally obtained, including SLC25A25-AS1, SNHG15, LOC283070, MALAT1, and NEAT1. Among these lncRNAs, MALAT1 has been suggested to play an important role in oxymatrine resistance in CRC and has the potential to be a therapeutic target and prognosis biomarker for CRC patients [54, 55]. NEAT1 was also reported as a promising circulating and prognosis biomarker for CRC [56, 57]. Also, Li et al. found that the decreasing expression levels of SLC25A25-AS1 promote cell proliferation and chemoresistance in CRC [58]. Other evidence showed the oncogenesis potential of SNHG15 and LOC283070 [59, 60].

Taken these results together, our analysis of downstream targets of miR-451a suggests its multiple roles in different stages of CRC, enhancing its potential and rationality to serve as a biomarker for CRC diagnosis.

### 3.4. KEGG Pathway and Gene Ontology Enrichments

**Figure 5:**
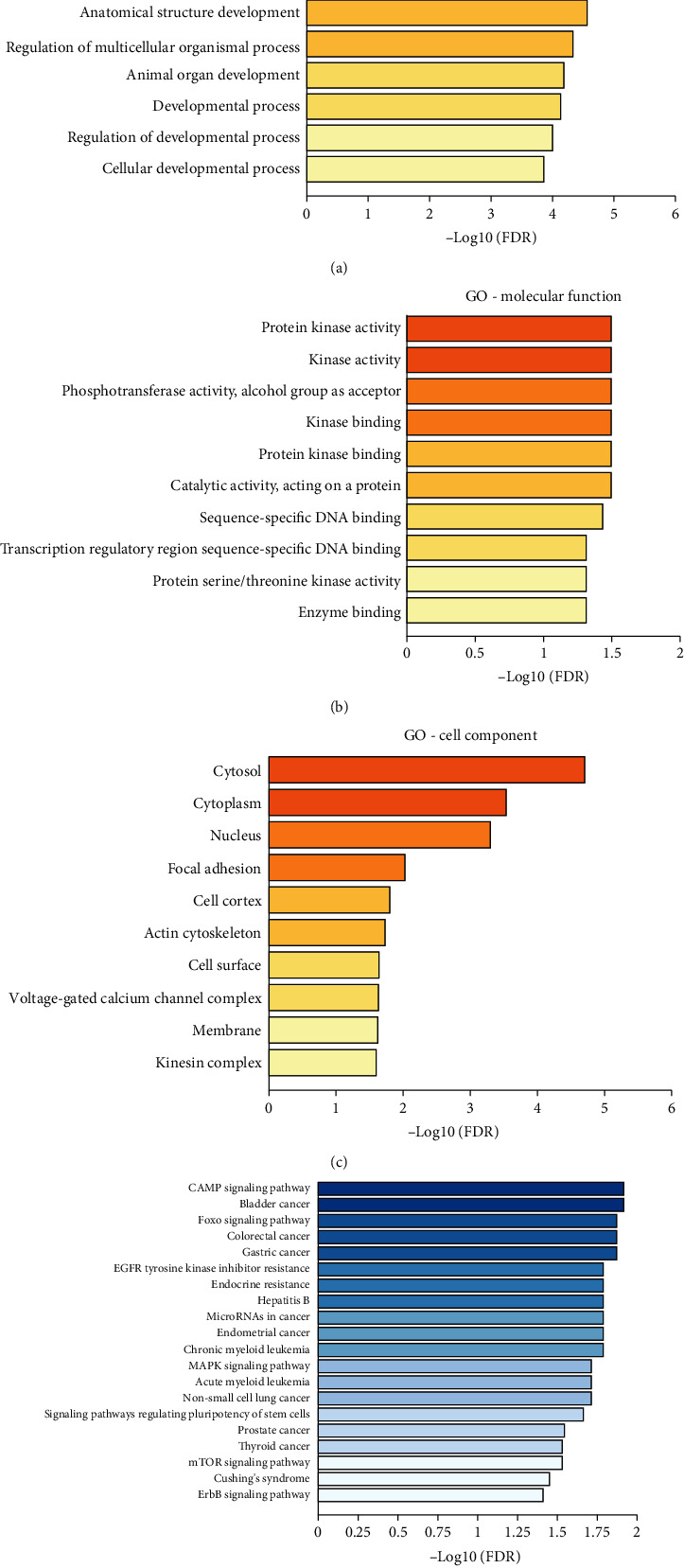
Results of functional enrichments. (a–c) Top 10 most significantly enriched items in Gene Ontology enrichments. (d) Top 20 most enriched pathways in KEGG enrichment.

We performed a functional enrichment analysis from the two different databases to investigate the mechanisms of miR-451a: KEGG pathway database and Gene Ontology database. The enrichment analysis was conducted using the STRING. The top 20 significantly enriched pathways and top 10 significantly enriched ontology items were selected at each level, as shown in Figure 5. The enriched GO items in BP included the positive regulation of cell process, developmental process, and organ development, suggesting that genes regulated by miR-451a may have positive regulations for cell development. This result further confirmed the suppressor role of miR-451a in CRC tissues. The regulation activity of miR-451a may happen in cytosol, cytoplasm, and nucleus, as supported by the results of GO items in CC. Results of GO items in MF, such as protein kinase activity, phosphotransferase activity, and kinase binding, indicated that genes regulated by miR-451a are strongly associated with protein activation; a set of study evidences support these results [61–63]. In KEGG pathway analysis, we observed that most of the top 20 pathways (about 70%) are related to CRC occurrence and development, including cAMP signaling pathway [64], FoxO signaling pathway [44], MAPK signaling pathway, and signaling pathways regulating pluripotency of stem cells [49, 65]. Notably, colorectal pathway ranked No. 4 in this enrichment, which enhanced our analysis confidence. Our enrichment study revealed the regulatory roles of miR-451a and the mechanisms it may be involved in. Thus, these data explained why miR-451a could serve as a promising biomarker for CRC diagnosis.

### 3.5. PPI Network Construction and Detection of Hub Nodes

**Figure 6:**
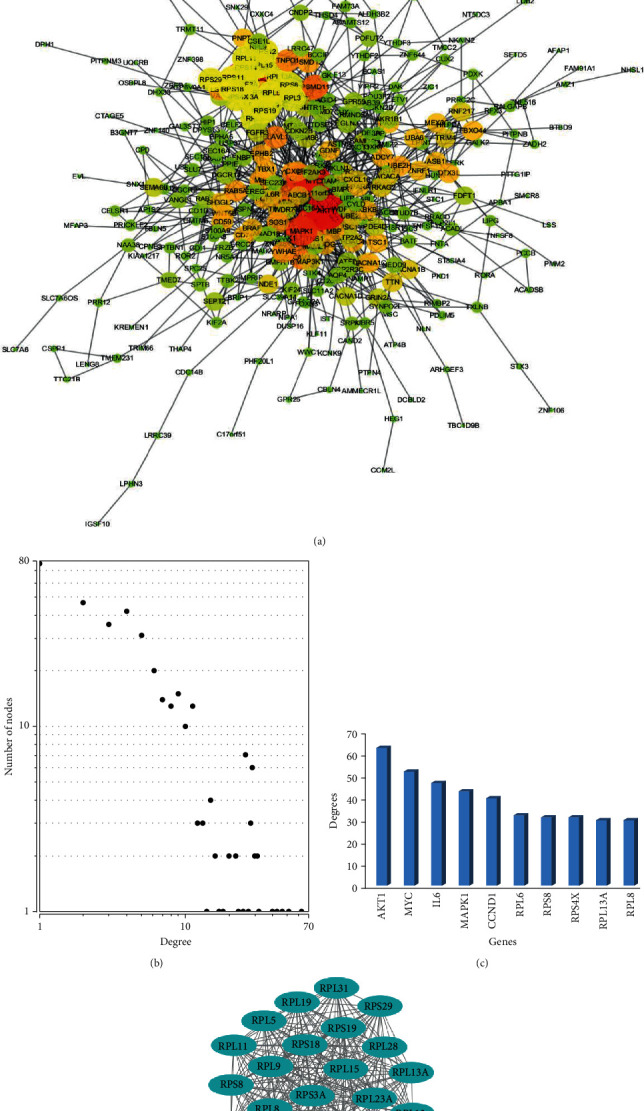
PPI network construction results. (a) The complete PPI network based on the interaction data from STRING. (b) Degree distribution of the nodes in the network. (c) Degree distribution of top 10 hub nodes. (d) Submodules with highest MCODE score.

Target mRNAs from both experimentally validated group and miRWalk predicted group (1163 mRNAs) were loaded into the STRING for PPI investigation. A total of 438 mRNAs were selected through this process for network construction. Results retrieved from STRING were processed and a PPI network for the target genes of miR-451a was visualized using the Cytoscape, as shown in Figure 6(a). The degree of a node reflects the number of connections with this node and the higher degree the node has, the more indispensable it will be for the stabilization of the network. Thus, degrees of these mRNAs were calculated and the top 10 hub nodes were screened, including AKT1, MYC, IL6, MAPK1, CCND1, RPL6, RPS8, RPS4X, RPL13A, and RPL8. The degree distributions of nodes in the network and the degree distributions of the top 10 hub nodes are presented in Figures 6(b) and 6(c). Interestingly, we noticed that these top 10 hub mRNAs could be roughly divided into four functional groups: AKT1 and MAPK1 are protein kinase coding genes; MYC and CCND1 are genes involved in cell cycle; RPL6, RPS8, RPS4X, and RPL13A are ribosomal protein coding genes, and IL6 is involved in T cell activation and tumor immune microenvironment modifications. In our gene expression analysis, we found that the expression of MYC and CCND1 was upregulated, with logFC of 1.3602 and 1.2455, respectively, and the expression of ribosomal proteins, such as RPL6, RPS8, and RPS4X, was slightly higher in CRC patients. However, the expression of IL6 showed no significant change. These results further showed the remarkable roles of miR-451a in CRC growth, inflammation, and differentiation.

Submodules were also detected using MCODE. The most significant module is presented in Figure 6(d). We noticed that most of the genes in this module are ribosomal protein coding genes. Emerging evidences have shown potentials of these proteins involving CRC carcinogenesis and drug targets [66–68]. These studies may give new insight into miR-451a function as it links with ribosomal proteins.

### 3.6. Diagnosis Potential of miR-451a

**Figure 7:**
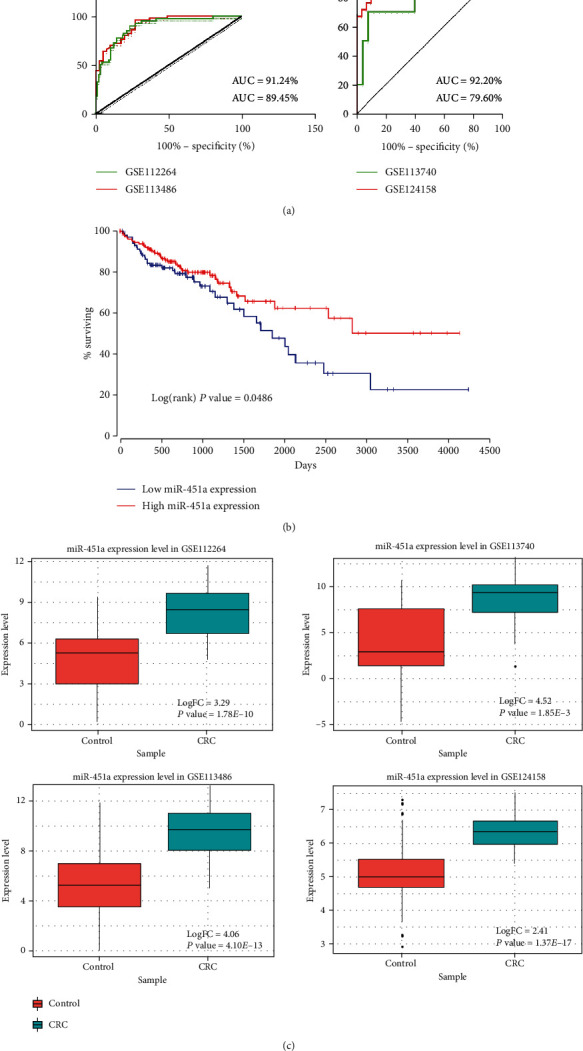
Results of reliability evaluation of miR-451a as a promising circulating CRC biomarker. (a) ROC curve of miR-451a in GSE112264, GSE113486 (these 2 datasets were used in previous biomarker identification part), GSE113740, and GSE124158 (these 2 datasets were new datasets obtained from GEO DataSets). (b) Survival rates of CRC patients. (c) Boxplots describing expression pattern of miR-451a in these 4 datasets.

Finally, we performed ROC curves and survival curves to test the discriminatory performance of miR-451a. These two graphs are shown in Figure 7. In ROC curves, totally 4 datasets were utilized here for validation. For GSE112264 and GSE113486 that we used for biomarker discovery, the values of area under curve (AUC) are 91.24% (CI: 0.857-0.968) and 89.45% (CI: 0.838-0.951), respectively. Besides, we also downloaded 2 new datasets from GEO DataSets, GSE113740 and GSE124158, to further confirm the biomarker reliability of miR-451a. The AUC values are 79.60% (CI: 0.614–0.978) and 92.2% (CI: 0.886–0.958), respectively. These consistent results indicate the favorable performance of miR-451a to distinguish CRC patient sera and normal sera. For survival curves, we obtained patients' information from TCGA and a total of 300 patients' data were used here. Patients were equally divided into low miR-451a expression group and high miR-451a expression group. The Kaplan–Meier analysis was used here for survival analysis. The results suggest that patients with high miR-451a expression have significantly higher survival rate compared to patients with low miR-451a expression (*P* value = 0.0486). These data show the tumor suppressor role of miR-451a in CRC patients, which are consistent with several previous findings of the role of miR-451a [69, 70]. These results give a strong support that miR-451a could evidently discriminate between CRC sera and normal sera, adding to previous evidences that pinpoint miR-451a as a diagnostic biomarker for CRC.

## 4. Discussion

To date, there are accumulating evidences that reveal various roles of miRNAs in the mechanisms of cancer. In clinical diagnosis, many studies demonstrated their brilliant performance in cancer detection and treatment due to their perfect biomarker characteristics [11]. However, previous studies mainly focus on dysregulated miRNAs in primary tumor, which have great contributions to expanding our understanding of the mechanisms of cancer but it provided limited knowledge for clinical diagnosis of cancer, especially for early diagnosis [71]. Hence, improvements in our current strategies for tumor screening are urgently required. In recent years, an increasing number of studies suggested that components of tumors are shed into the blood circulation, which could be detected from body liquids. These findings improve many aspects of tumor screening and management and give researchers new insights for the methods for early detection of cancer.

In this study, we used publicly available microarray data and miRNA-seq data from GEO DataSets and TCGA data portal for integrative bioinformatics analysis strategy to identify novel circulating miRNA biomarkers for CRC diagnosis. We considered miRNAs as candidate circulating biomarkers under at least two essential characteristics: first, they are dysregulated in CRC sera compared with normal sera; second, they are sufficiently powerful to indicate the status of health and disease. To address these two characteristics, we selected DE-genes at first. Then, a specific tool named miRNA-BD was used to generate the candidate miRNA biomarkers using the inputs of DE-genes. This tool is based on the Pipeline of Outlier MicroRNA Analysis (POMA) algorithm, which has been validated in many other complex diseases, such as pediatric acute myeloid leukemia and autism [8, 72]. At the same time, we retrieved serum miRNA expression microarray data and primary tumor miRNA-seq data to check the DE-miRNAs in both CRC serums and primary tumors. Taken these data together, we made an overlap and finally miR-451a was selected as the candidate CRC biomarker. An interesting expression difference was noticed in the expression analysis, whereby miR-451a is upregulated in CRC sera but downregulated in primary tumors, indicating a relevant pathway signaling and gene expression changed from primary tumors to human sera.

To further investigate the functional roles of miR-451a, we performed various downstream analysis of miR-451a, including target identification, functional enrichment, and PPI network analysis. In target identification, we noticed that 20 out of 31 validated gene targets were reported to be involved in CRC occurrence and development, most of which are associated with cancer cell proliferation and differentiation [45, 48, 63]. Meanwhile, most of the predicted gene targets through miRWalk are also relevant to CRC. We returned to check the expression pattern in our microarray analysis and found upregulations of these genes, suggesting its role in tumor suppression role in CRC primary tumors. We also examined the lncRNA targets of miR-451a in which 41 lncRNA targets were obtained after overlapping the results from experimentally validated targets and computationally predicted targets. Here, we observed that 5 of the targets were previously demonstrated to have contributions to CRC drug resistance and prognosis. Results of pathway and GO enrichment analysis provided additional evidences for the functions of these targets. These target identification results illustrate the general functional pattern of miR-451a.

We also performed PPI network analysis to reveal the correlations among these target genes of miR-451a. Through PPI network construction, a series of hub genes were detected. Most significant hub genes are mainly enriched on protein kinases, ribosomal protein, cell cycle regulation, and tumor immune microenvironment modifications. We also identified submodules of this network, and the module with highest MOCDE score showed a potential role of miR-451a in regulating ribosomal protein coding, although the expression of these target genes in our microarray analysis is not significantly discriminative.

Finally, we measured the biomarker robustness of miR-451a through ROC curve analysis and survival analysis. The results of ROC curve showed high sensitivity of miR-451a, with 91.24% and 89.45% AUC values in two sera microarray datasets. The survival analysis also exhibited a significant distinguishing pattern between the high miR-451a expression group and the low miR-451a expression group.

Taken these results together, we managed to illustrate the functional pattern of miR-451a at a systematic level and identified it as a potential circulating biomarker for CRC. However, the dynamic and complexity of CRC required further confirmations of miR-451a through clinical trials, specifically to elucidate the change in expression levels of miR-451a from the primary tumor to serum. Detailed mechanism research of this change should also be conducted in the future.

## 5. Conclusions

In conclusion, we identified the significance of miR-451a in CRC. Using integrative data mining and bioinformatics analysis, we explained why miR-451a is an excellent circulating biomarker for early CRC diagnosis. Studies with large biological and clinical data or studies with detailed biological experiments should be carried out to confirm the critical role of miR-451a in CRC.

## Figures and Tables

**Table 1:** Summary of microarray datasets for this study.

GEO accession	Gene/miRNA	Source	Platforms	Number of samples		Ref.
				CRC	Normal	
GSE41258	Gene	Tissue	Affymetrix Human Genome U133A Array	186	54	[15, 16]
GSE112264	miRNA	Serum	3D-Gene Human miRNA V21_1.0.0	50	41	[17]
GSE113486	miRNA	Serum	3D-Gene Human miRNA V20_1.0.0	40	100	[18]

## Data Availability

The figure and table data used to support the findings of this study are included within the article.

## References

[1] Siegel R. L., Miller K. D., Jemal A. (2018). Cancer statistics, 2018. *CA: a Cancer Journal for Clinicians*.

[2] Hofsli E., Sjursen W., Prestvik W. S. (2013). Identification of serum microRNA profiles in colon cancer. *British Journal of Cancer*.

[3] Duffy M. J. (2008). Clinical uses of tumor markers: a critical review. *Critical Reviews in Clinical Laboratory Sciences*.

[4] Swiderska M., Choromańska B., Dąbrowska E. (2014). The diagnostics of colorectal cancer. *Contemp Oncol (Pozn)*.

[5] Zhang X., Sun X. F., Cao Y. (2018). CBD: a biomarker database for colorectal cancer. *Database: The Journal of Biological Databases and Curation*.

[6] Niedzwiecki D., Hasson R. M., Lenz H. J. (2017). A study of thymidylate synthase expression as a biomarker for resectable colon cancer: alliance (cancer and leukemia group B) 9581 and 89803. *The Oncologist*.

[7] Ge Q., Zhou Y., Lu J., Bai Y., Xie X., Lu Z. (2014). miRNA in plasma exosome is stable under different storage conditions. *Molecules*.

[8] Shen L., Lin Y., Sun Z., Yuan X., Chen L., Shen B. (2016). Knowledge-guided bioinformatics model for identifying autism spectrum disorder diagnostic microRNA biomarkers. *Scientific Reports*.

[9] Zhou Q., Huang S. X., Zhang F. (2017). MicroRNAs: a novel potential biomarker for diagnosis and therapy in patients with non-small cell lung cancer. *Cell Proliferation*.

[10] Chen X., Ba Y., Ma L. (2008). Characterization of microRNAs in serum: a novel class of biomarkers for diagnosis of cancer and other diseases. *Cell Research*.

[11] Garzon R., Calin G. A., Croce C. M. (2009). MicroRNAs in cancer. *Annual Review of Medicine*.

[12] Sun W., Wang X., Li J. (2018). MicroRNA-181a promotes angiogenesis in colorectal cancer by targeting SRCIN1 to promote the SRC/VEGF signaling pathway. *Cell Death & Disease*.

[13] Jin Y., Cheng H., Cao J., Shen W. (2019). MicroRNA 32 promotes cell proliferation, migration, and suppresses apoptosis in colon cancer cells by targeting OTU domain containing 3. *Journal of Cellular Biochemistry*.

[14] Griffiths-Jones S., Saini H. K., van Dongen S., Enright A. J. (2008). miRBase: tools for microRNA genomics. *Nucleic Acids Research*.

[15] Sheffer M., Bacolod M. D., Zuk O. (2009). Association of survival and disease progression with chromosomal instability: a genomic exploration of colorectal cancer. *Proceedings of the National Academy of Sciences of the United States of America*.

[16] Martin M. L., Zeng Z., Adileh M. (2018). Logarithmic expansion of LGR5(+) cells in human colorectal cancer. *Cellular Signalling*.

[17] Urabe F., Matsuzaki J., Yamamoto Y. (2019). Large-scale circulating microRNA profiling for the liquid biopsy of prostate cancer. *Clinical Cancer Research*.

[18] Usuba W., Urabe F., Yamamoto Y. (2019). Circulating miRNA panels for specific and early detection in bladder cancer. *Cancer Science*.

[19] Smyth G. K., Gentleman R. (2005). *Limma: linear models for microarray data, in Bioinformatics and Computational Biology Solutions Using R and Bioconductor*.

[20] Robinson M. D., McCarthy D. J., Smyth G. K. (2009). edgeR: a bioconductor package for differential expression analysis of digital gene expression data. *Bioinformatics*.

[21] Lin Y., Wu W., Sun Z., Shen L., Shen B. (2018). MiRNA-BD: an evidence-based bioinformatics model and software tool for microRNA biomarker discovery. *RNA Biology*.

[22] Hsu S. D., Lin F. M., Wu W. Y. (2011). miRTarBase: a database curates experimentally validated microRNA-target interactions. *Nucleic Acids Research*.

[23] Dweep H., Gretz N. (2015). miRWalk2.0: a comprehensive atlas of microRNA-target interactions. *Nature Methods*.

[24] John B., Enright A. J., Aravin A., Tuschl T., Sander C., Marks D. S. (2004). Human microRNA targets. *PLoS Biology*.

[25] Miranda K. C., Huynh T., Tay Y. (2006). A pattern-based method for the identification of microRNA binding sites and their corresponding heteroduplexes. *Cell*.

[26] Agarwal V., Bell G. W., Nam J. W., Bartel D. P. (2015). Predicting effective microRNA target sites in mammalian mRNAs. *eLife*.

[27] Paraskevopoulou M. D., Georgakilas G., Kostoulas N. (2013). DIANA-LncBase: experimentally verified and computationally predicted microRNA targets on long non-coding RNAs. *Nucleic Acids Research*.

[28] Gao Y., Wang P., Wang Y. (2019). Lnc2Cancer v2.0: updated database of experimentally supported long non-coding RNAs in human cancers. *Nucleic Acids Research*.

[29] Szklarczyk D., Gable A. L., Lyon D. (2019). STRING v11: protein-protein association networks with increased coverage, supporting functional discovery in genome-wide experimental datasets. *Nucleic Acids Research*.

[30] Kanehisa M., Goto S., Sato Y., Furumichi M., Tanabe M. (2011). KEGG for integration and interpretation of large-scale molecular data sets. *Nucleic Acids Research*.

[31] Chin C. H., Chen S. H., Wu H. H., Ho C. W., Ko M. T., Lin C. Y. (2014). cytoHubba: identifying hub objects and sub-networks from complex interactome. *BMC Systems Biology*.

[32] Sowden J., Leigh S., Talbot I., Delhanty J., Edwards Y. (1993). Expression from the proximal promoter of the carbonic anhydrase 1 gene as a marker for differentiation in colon epithelia. *Differentiation*.

[33] van de Wetering M., Sancho E., Verweij C. (2002). The *β*-Catenin/TCF-4 complex imposes a crypt progenitor phenotype on colorectal cancer cells. *Cell*.

[34] Ghaleb A. M., McConnell B. B., Kaestner K. H., Yang V. W. (2011). Altered intestinal epithelial homeostasis in mice with intestine-specific deletion of the Krüppel-like factor 4 gene. *Developmental Biology*.

[35] Offermans N. S. M., Ketcham S. M., van den Brandt P. A., Weijenberg M. P., Simons C. C. J. M. (2018). Alcohol intake, ADH1B and ADH1C genotypes, and the risk of colorectal cancer by sex and subsite in the Netherlands Cohort Study. *Carcinogenesis*.

[36] Pattison A. M., Merlino D. J., Blomain E. S., Waldman S. A. (2016). Guanylyl cyclase C signaling axis and colon cancer prevention. *World Journal of Gastroenterology*.

[37] Shangkuan W. C., Lin H. C., Chang Y. T. (2017). Risk analysis of colorectal cancer incidence by gene expression analysis. *PeerJ*.

[38] Kumamoto K., Nakachi Y., Mizuno Y. (2019). Expressions of 10 genes as candidate predictors of recurrence in stage III colon cancer patients receiving adjuvant oxaliplatin-based chemotherapy. *Oncology Letters*.

[39] Chai B., Guo Y., Cui X. (2019). MiR-223-3p promotes the proliferation, invasion and migration of colon cancer cells by negative regulating PRDM1. *American Journal of Translational Research*.

[40] Kim C. W., Oh E. T., Kim J. M. (2018). Hypoxia-induced microRNA-590-5p promotes colorectal cancer progression by modulating matrix metalloproteinase activity. *Cancer Letters*.

[41] Mamoori A., Wahab R., Vider J., Gopalan V., Lam A. K. Y. (2019). The tumour suppressor effects and regulation of cancer stem cells by macrophage migration inhibitory factor targeted miR-451 in colon cancer. *Gene*.

[42] ZHOU H. U. A. N., HU Y. U., WANG W. E. I. P. E. N. G. (2015). Expression of Oct-4 is significantly associated with the development and prognosis of colorectal cancer. *Oncology Letters*.

[43] Valverde A., Peñarando J., Cañas A. (2015). Simultaneous inhibition of EGFR/VEGFR and cyclooxygenase-2 targets stemness-related pathways in colorectal cancer cells. *PLoS One*.

[44] Li Y., Wang J., Dai X. (2015). miR-451 regulates FoxO3 nuclear accumulation through Ywhaz in human colorectal cancer. *American Journal of Translational Research*.

[45] Debebe Z., Rathmell W. K. (2015). Ror2 as a therapeutic target in cancer. *Pharmacology & Therapeutics*.

[46] Voloshanenko O., Schwartz U., Kranz D. (2018). *β*-catenin-independent regulation of Wnt target genes by RoR2 and ATF2/ATF4 in colon cancer cells. *Scientific Reports*.

[47] Lara E., Calvanese V., Huidobro C. (2010). Epigenetic repression of ROR2 has a Wnt-mediated, pro-tumourigenic role in colon cancer. *Molecular Cancer*.

[48] Yang Y., Li X. J., Li P., Guo X. T. (2018). MicroRNA-145 regulates the proliferation, migration and invasion of human primary colon adenocarcinoma cells by targeting MAPK1. *International Journal of Molecular Medicine*.

[49] Fang J. Y., Richardson B. C. (2005). The MAPK signalling pathways and colorectal cancer. *The Lancet Oncology*.

[50] Ghoshal K., Motiwala T., Claus R. (2010). HOXB13, a target of DNMT3B, is methylated at an upstream CpG island, and functions as a tumor suppressor in primary colorectal tumors. *PLoS One*.

[51] Chen S., Yue T., Huang Z. (2019). Inhibition of hydrogen sulfide synthesis reverses acquired resistance to 5-FU through miR-215-5p-EREG/TYMS axis in colon cancer cells. *Cancer Letters*.

[52] Luo Y., Xie C., Brocker C. N. (2019). Intestinal PPAR*α* protects against colon carcinogenesis via regulation of methyltransferases DNMT1 and PRMT6. *Gastroenterology*.

[53] Du Q., Guo X., Zhang X. (2016). SYNJ2 Variant Rs9365723 is Associated with Colorectal Cancer Risk in Chinese Han Population. *The International Journal of Biological Markers*.

[54] Xiong Y., Wang J., Zhu H., Liu L., Jiang Y. (2018). Chronic oxymatrine treatment induces resistance and epithelial‑mesenchymal transition through targeting the long non-coding RNA MALAT1 in colorectal cancer cells. *Oncology Reports*.

[55] Zheng H. T., Shi D. B., Wang Y. W. (2014). High expression of lncRNA MALAT1 suggests a biomarker of poor prognosis in colorectal cancer. *International Journal of Clinical and Experimental Pathology*.

[56] Peng W., Wang Z., Fan H. (2017). LncRNA NEAT1 impacts cell proliferation and apoptosis of colorectal cancer via regulation of Akt signaling. *Pathology Oncology Research*.

[57] Wu Y., Yang L., Zhao J. (2015). Nuclear-enriched abundant transcript 1 as a diagnostic and prognostic biomarker in colorectal cancer. *Molecular Cancer*.

[58] Li Y., Huang S., Li Y. (2016). Decreased expression of LncRNA SLC25A25-AS1 promotes proliferation, chemoresistance, and EMT in colorectal cancer cells. *Tumour Biology*.

[59] Wang Z. L., Li B., Piccolo S. R. (2016). Integrative analysis reveals clinical phenotypes and oncogenic potentials of long non-coding RNAs across 15 cancer types. *Oncotarget*.

[60] Wang L., Lin Y., Meng H. (2016). Long non-coding RNA LOC283070 mediates the transition of LNCaP cells into androgen-independent cells possibly via CAMK1D. *American Journal of Translational Research*.

[61] Sundram V., Ganju A., Hughes J. E., Khan S., Chauhan S. C., Jaggi M. (2014). Protein kinase D1 attenuates tumorigenesis in colon cancer by modulating *β*-catenin/T cell factor activity. *Oncotarget*.

[62] Wang J., Wu H. F., Shen W. (2016). SRPK2 promotes the growth and migration of the colon cancer cells. *Gene*.

[63] Slattery M. L., Lundgreen A., Wolff R. K. (2012). MAP kinase genes and colon and rectal cancer. *Carcinogenesis*.

[64] Tse C. M., Yin J., Singh V. (2019). cAMP stimulates SLC26A3 activity in human colon by a CFTR-dependent mechanism that does not require CFTR activity. *Cellular and Molecular Gastroenterology and Hepatology*.

[65] Bitarte N., Bandres E., Boni V. (2011). MicroRNA-451 is involved in the self-renewal, tumorigenicity, and chemoresistance of colorectal cancer stem cells. *Stem Cells*.

[66] Dong Z., Jiang H., Liang S., Wang Y., Jiang W., Zhu C. (2019). Ribosomal protein L15 is involved in colon carcinogenesis. *International Journal of Medical Sciences*.

[67] Mushtaq M., Ali R. H., Kashuba V., Klein G., Kashuba E. (2016). S18 family of mitochondrial ribosomal proteins: evolutionary history and Gly132 polymorphism in colon carcinoma. *Oncotarget*.

[68] Boyle K. A., van Wickle J., Hill R. B., Marchese A., Kalyanaraman B., Dwinell M. B. (2018). Mitochondria-targeted drugs stimulate mitophagy and abrogate colon cancer cell proliferation. *The Journal of Biological Chemistry*.

[69] Wang R., Wang Z. X., Yang J. S., Pan X., de W., Chen L. B. (2011). MicroRNA-451 functions as a tumor suppressor in human non-small cell lung cancer by targeting ras-related protein 14 (RAB14). *Oncogene*.

[70] Vidal D. O., Ramão A., Pinheiro D. G. (2018). Highly expressed placental miRNAs control key biological processes in human cancer cell lines. *Oncotarget*.

[71] Marcuello M., Vymetalkova V., Neves R. P. L. (2019). Circulating biomarkers for early detection and clinical management of colorectal cancer. *Molecular Aspects of Medicine*.

[72] Yan W., Xu L., Sun Z. (2015). MicroRNA biomarker identification for pediatric acute myeloid leukemia based on a novel bioinformatics model. *Oncotarget*.

